# *E. coli* bacteraemia and antimicrobial resistance following antimicrobial prescribing for urinary tract infection in the community

**DOI:** 10.1186/s12879-022-07768-7

**Published:** 2022-10-28

**Authors:** C McCowan, A Bakhshi, A McConnachie, W Malcolm, Barry SJE, Virginia Hernandez Santiago, A Leanord

**Affiliations:** 1 School of Medicine, University of St Andrews, Fife, Scotland; 2 University of West of Scotland, Paisley, Scotland; 3 Robertson Centre for Biostatistics Institute of Health and Wellbeing, University of Glasgow, Glasgow, Scotland; 4 ARHAI Scotland, NHS National Services, Edinburgh, Scotland; 5 Department of Mathematics and Statistics, University of Strathclyde, Glasgow, Scotland; 6 Institute of Infection, Immunity and Inflammation, University of Glasgow, Glasgow, Scotland; 7 Division of Population and Behavioural Sciences, School of Medicine, University of St Andrews. North Haugh, KY16 9TF St Andrews, UK

**Keywords:** *Escherichia coli*, Bacteraemia, Bloodstream infection, Antimicrobial resistance, Epidemiology

## Abstract

**Background:**

Urinary tract infections are one of the most common infections in primary and secondary care, with the majority of antimicrobial therapy initiated empirically before culture results are available. In some cases, however, over 40% of the bacteria that cause UTIs are resistant to some of the antimicrobials used, yet we do not know how the patient outcome is affected in terms of relapse, treatment failure, progression to more serious illness (bacteraemia) requiring hospitalization, and ultimately death. This study analyzed the current patterns of antimicrobial use for UTI in the community in Scotland, and factors for poor outcomes.

**Objectives:**

To explore antimicrobial use for UTI in the community in Scotland, and the relationship with patient characteristics and antimicrobial resistance in *E. coli* bloodstream infections and subsequent mortality.

**Methods:**

We included all adult patients in Scotland with a positive blood culture with *E. coli* growth, receiving at least one UTI-related antimicrobial (amoxicillin, amoxicillin/clavulanic acid, ciprofloxacin, trimethoprim, and nitrofurantoin) between 1st January 2009 and 31st December 2012. Univariate and multivariate logistic regression analysis was performed to understand the impact of age, gender, socioeconomic status, previous community antimicrobial exposure (including long-term use), prior treatment failure, and multi-morbidity, on the occurrence of *E. coli* bacteraemia, trimethoprim and nitrofurantoin resistance, and mortality.

**Results:**

There were 1,093,227 patients aged 16 to 100 years old identified as receiving at least one prescription for the 5 UTI-related antimicrobials during the study period. Antimicrobial use was particularly prevalent in the female elderly population, and 10% study population was on long-term antimicrobials. The greatest predictor for trimethoprim resistance in *E. coli* bacteraemia was increasing age (OR 7.18, 95% CI 5.70 to 9.04 for the 65 years old and over group), followed by multi-morbidity (OR 5.42, 95% CI 4.82 to 6.09 for Charlson Index 3+). Prior antimicrobial use, along with prior treatment failure, male gender, and higher deprivation were also associated with a greater likelihood of a resistant *E. coli* bacteraemia. Mortality was significantly associated with both having an *E. coli* bloodstream infection, and those with resistant growth.

**Conclusion:**

Increasing age, increasing co-morbidity, lower socioeconomic status, and prior community antibiotic exposure were significantly associated with a resistant *E. coli* bacteraemia, which leads to increased mortality.

**Supplementary Information:**

The online version contains supplementary material available at 10.1186/s12879-022-07768-7.

## Introduction

Urinary tract infection (UTI) is the second most common clinical indication for empirical antimicrobial treatment in primary and secondary care, and is the most frequently occurring health care associated infection in the UK National Health System (NHS) [1]. Antimicrobial resistance has been recognised as a concern for the future treatment of infections, leading to repeat prescriptions, continued symptoms, complications, increased use of broad spectrum antimicrobials [2], and mortality.

There is a direct relationship between the levels of antimicrobial prescribed and the level of resistance seen in a range of organisms [3]. However, the relationship between antimicrobial consumption and development of resistance can be complex [4]. At a local level, the correlation between antimicrobial use in the community and resistance is well recognised [5–9], but the correlation between reductions in community prescribing and reductions in resistance are potentially imbalanced. Gram-positive resistance has been reported to decrease in response to primary care stewardship, but the link between reduced antimicrobial use and lower resistance in Gram-negative bacteria is less consistent [10, 11]. Prescribing data for UTIs shows that a large reduction of (20%) antimicrobial use in the community correlated to only a small (1%) decrease in resistance to amoxicillin or amoxicillin/clavulanic acid, with similar sized effects seen for trimethoprim usage and resistance [12–14]. These small reductions in resistance, despite large reductions in prescribing, are highly dependent on the genetic profile of the organisms, how the resistance determinants are carried and spread due to co-selection by other drugs and the level of bacterial fitness cost [15]. The stability of mobile genetic elements (plasmids and integrons) carrying resistance genes is a major factor in the population dynamics of the resistant and sensitive bacterial populations. As a result of the above each drug-bug combination will react very differently to changes/restrictions in the drug use [13, 16].

At the patient level, individuals prescribed an antimicrobial for a UTI are prone to develop resistant infections, with resistance persisting for up to 12 months after the cessation of antimicrobial [6, 17, 18]. The time necessary to develop resistance has been shown to be inversely correlated with the amount of antimicrobial given [11]. Likewise, multiple prescriptions and longer durations of treatment showed increased rates of resistance [6]. The correlation between the number of prescriptions and resistance has been quantified in recent work where multiple prescriptions increased the risk of a resistant *Escherichia coli* (*E. coli)* by two to three fold compared to a single course of treatment [19]. Recent work examining *E. coli* urine isolates showed that having a larger number of different antimicrobial prescriptions (more than 4) in the previous six months and also increasing Defined Daily Doses (DDDs) were associated with increased risk of resistance and multiple drug resistance [20].

Community use represents the bulk, approximately 80%, of antimicrobial prescriptions within the UK [7], with up to a third considered to be inappropriate [21], mostly consisting of broad-spectrum antimicrobials [22]. Many of these antimicrobials are prescribed for the treatment of UTIs [23–29]. Five antimicrobials (amoxicillin, ciprofloxacin, amoxicillin/clavulanic acid, nitrofurantoin and trimethoprim) make up approximately 70-80% of the antimicrobials prescribed for the treatment of UTIs. The majority of cases of antimicrobial therapy for UTI are also initiated empirically before culture results are available [30], as urine cultures are not routinely recommended in certain patient populations (e.g. non-pregnant women) [31]. In some cases over 40% of the bacteria that cause UTIs are resistant to some of the antimicrobials used [29] with this increasing risk for relapse and treatment failure [32, 33]. *E. coli* is the most common cause of bacteraemia in high-income countries, and the burden of *E. coli* bacteremia is substantial, especially among the elderly [34]. To enable the implementation of effective mitigation and prevention strategies, we need to understand better the epidemiology and risk factors of invasive *E. coli* infections. There is growing evidence showing that increases in the rates of *E. coli* bacteraemia are being driven predominantly by community-onset infections [35], particularly infections of the urinary tract [34], yet we do not know how and which specific factors increase the likelihood for progression to more serious illness requiring hospitalisation, and ultimately death, after treatment for UTI in the community.

This paper describes the epidemiology of the use of trimethoprim, nitrofurantoin, amoxicillin/clavulanic acid, amoxicillin and ciprofloxacin for UTI in the community across Scotland, characterises the population using these antimicrobials and patterns of use, and examines how prior use of antimicrobials is associated with antimicrobial resistance in bloodstream infections and deaths.

## Methods

### Data linkage

The NHS National Services Scotland (NSS) Prescribing Information System (PIS) [36] holds all community dispensed prescriptions in NHS Scotland at an individual patient level and was examined to identify a cohort of patients who had been dispensed a UTI-related antimicrobial (amoxicillin, ciprofloxacin, amoxicillin/clavulanic acid, nitrofurantoin or trimethoprim) at any point from 1st January 2009 to 31st December 2012. Entry to the cohort was defined as the first prescription in the study period for one of the UTI-related antimicrobials. Gender and age at cohort entry were recorded for all patients. Only adults (age range 16–100 years old) were included in the study. Socio-economic status based on home postcode at entry to the cohort was measured by Scottish Index of Multiple Deprivation quintiles [37]. The Scottish Index of Multiple Deprivation (SIMD) is a relative measure of deprivation used by the Scottish Government to define socioeconomic deprivation across small geographical areas (also known as datazones), based on income, employment, education, health, access to services, crime and housing. Geographical areas are categorised into five quintiles, with quintile 1 being the most deprived, and quintile 5 the most affluent. Prescribing data for all the UTI-related antimicrobial prescriptions dispensed in the study period was linked to reports of all bacteraemia from the Electronic Communication of Surveillance in Scotland (ECOSS) data, and to patient level data on hospital admission from NSS General/Acute Inpatient and Day Case (SMR01) data and deaths from National Registrar Scotland (NRS) data. A universal patient registration system in Scotland uses a unique 10-digit identifier called the community health index number (CHI) which allows data linkage of all NHS encounters. The extraction and linkage were performed by the electronic Data Research and Innovation Service (eDRIS) [38] with all identifiers removed and replaced with a project specific pseudonymous identifier and access to the data provided via a Safe Haven secure analytical platform. No identifiable data was accessed by the research team.

In addition to demographic characteristics, potential risk factors associated with the presence of *E. coli* bacteraemia and antimicrobial resistance were examined using patterns of antimicrobial prescribing, prior hospitalisations and co-morbidity.

Patient level antimicrobial use data were used to identify how many prescriptions of each agent they received within each year in the study period. We also calculated total DDDs for each antimicrobial, defined as the assumed average maintenance dose per day for an individual drug’s main indication in adults. Patients who had received the same antimicrobial six or more times within any rolling 12-month period were defined as long-term users. Patients who received a different antimicrobial within 60 days were classed as having had a treatment failure as we assumed this was used to treat the same infection.

A Charlson Index of comorbidity [39] was calculated based on diagnostic ICD-10 codes from hospital discharge records. Previous admissions to hospital were also identified from these records including whether a High Dependency Unit (HDU) or Intensive Care Unit (ICU) stay was part of the admission.

Presence of any organism was identified through at least one record of bacteraemia for the patient within the ECOSS dataset. Reporting of urinary isolates for *E. coli* to ECOSS is not mandatory and so whilst these were initially examined they were not included within any analysis as there was only a sample of records available. *E. coli* bacteraemia related to blood samples, for which mandatory reporting across Scotland exists, was examined and reports on whether the isolate causing bacteraemia was sensitive or resistant to each of the tested antimicrobials was recorded (using Clinical and Laboratory Standard Institute [CLSI] recommended minimum inhibitory concentrations [40]). Each isolate was tested against several different antimicrobials and susceptibility was reported for any, including trimethoprim and nitrofurantoin, which are used primarily for treatment of urinary tract infection (referred to as UTI-specific antimicrobials).

### Statistical analysis

Demographic characteristics, long-term users, treatment failures and number of antimicrobials dispensed were summarised for patients with any prescriptions, in total and by clinically defined group of UTI-related antimicrobials. The proportions of patients with bacteraemia, based on a positive blood culture for *E. coli*, were summarised across the groups for all of these variables and compared between the groups using chi-squared tests. Similarly, the proportions of patients who died within the study period were summarised and compared across demographic and usage groups using a chi-squared test for differences in proportions. Two-sample t-tests were used for comparison of continuous variables.

The main outcome measures were (a) first occurrence of positive *E. coli* bacteraemia, and (b) first occurrence of resistance to trimethoprim within the study period. Potential predictors of antimicrobial resistance were based on patterns of antimicrobial prescribing (e.g. long-term use, cumulative dose defined as DDDs, treatment failure). For each patient, these predictor variables are time-varying covariates, i.e. they change throughout the follow-up period. The standard analysis approach for incident events is survival analysis (e.g. Cox regression) potentially with time-varying covariates. However, due to the size of the dataset we used a partial logistic regression for survival analysis method [41]. We divided the follow-up period into fixed distinct time intervals, to allow for a simplified analysis via logistic regression, with the outcome being the first occurrence of an event during the time interval given that subjects were event free at the start of the interval, and predictors being based on each patient’s status at the start of the interval. We divided the study period into six-month intervals and used information from 2010 to 2012 for the modelling. The initial two intervals for 2009 were used to define patients’ status at the beginning of 2010 but no modelling for outcomes was performed over this time period. Hence the modelling was done using six intervals (two intervals per year for three years) with all events each study period modelled as a risk over the next 6-months (180 days).

We developed univariable models associating the two outcomes with each of the predictors and then created a final multivariable model for statistically significant predictors (selected in univariate analysis significant at p < 0.05) using manual stepwise selection. All analyses were carried out using the Statistical software R version 3.4.0 [42].

## Results

### Demographic characteristics

There were 1,093,227 patients aged 16 to 100 years old identified as receiving at least one prescription for the 5 UTI-related antimicrobials between 1 and 2009 and 31 December 2012 (amoxicillin, ciprofloxacin, amoxicillin/clavulanic acid, nitrofurantoin and trimethoprim - see Table 1). Over two-thirds of the cohort were female, with almost a third of all patients being older than 65 years, and patients tended to be relatively socioeconomically deprived, with almost half of the population (44.2%) belonging to the two most deprived quintiles. Almost half of all patients (44.9%) were prescribed one type of UTI-related antimicrobial during the study period, while just over a third (36.8%) were prescribed two different antimicrobials and 18% had 3 or more different antimicrobials prescribed. When examining the UTI-specific antimicrobials (nitrofurantoin and trimethoprim) only, there was also a higher proportion of women (615,027–81.8%) and patients aged older than 65 years (254,784 − 33.9%) with 14.8% (161,787 patients) receiving both agents within the same year.

**Table 1:** Characteristics of patients with at least one UTI-related antimicrobial prescribed

Antimicrobial Group	Amoxicillin, ciprofloxacin, amoxicillin/clavulanic acid, nitrofurantoin or trimethoprim	Nitrofurantoin or trimethoprim
**Any**	**1,093,227 (100.0%)**	**752,225 (68.8%)**
**Gender**		
** Male** ** Female**	310,974 (28.4%) 782,253 (71.6%)	137,198 (18.2%) 615,027 (81.8%)
**Age group**		
** 16-34** ** 35-49** ** 50-64** ** > 65**	268,896 (24.6%) 242,173 (22.2%) 247,287 (22.6%) 334,871 (30.6%)	184,476 (24.5%) 152,857 (20.3%) 160,108 (21.3%) 254,784 (33.9%)
**SIMD quintile (Q1 = Most Deprived, Q5 = Least Deprived)**		
** Q1** ** Q2** ** Q3** ** Q4** ** Q5**	247,671 (22.8%) 232,451 (21.4%) 215,015 (19.8%) 198,832 (18.3%) 190,210 (17.5%)	169,301 (22.7%) 159,286 (21.4%) 147,401 (19.8%) 138,783 (18.6%) 131,272 (17.6%)
**Long-term users of any antimicrobials**		
Yes	85,036 (7.8%)	32,968 (4.4%)
No	1,008,191 (92.2%)	719,257 (95.6%)
**Treatment failures**		
	195,184 (17.9%)	Trimethoprim: 72,705 (11.2%) Nitrofurantoin: 38,625 (16.7%)
**Number of antimicrobials received per patient**		
** 1** ** 2** ** 3** ** 4** ** 5**	491,315 (44.9%) 402,731 (36.8%) 144,671 (13.2%) 44,179 (4.0%) 10,331 (0.9%)	590,438 (54.0%) 161,787 (14.8%)

### Long-term users and treatment failures

Prescribing patterns showed that 85,036 (7.8%) of patients were long-term users of antimicrobials (any antimicrobial); and there were 32,968 long-term users (4.4%) of UTI-specific antimicrobials (defined as patients that had 6 or more prescriptions for the same antimicrobial in any 12-month period).

There were 195,184 (17.9%) patients classed as having a treatment failure (two different antimicrobials prescribed within a 60-day period).

For the UTI-specific antimicrobials, 11.2% of all patients (72,705) prescribed trimethoprim had a treatment failure, while 16.7% (38,625) of those prescribed nitrofurantoin had a treatment failure (Table 1).

### ***E. coli*** bacteraemia

Of the total sample of 1,093,227 patients, 121,716 (11.1%) patients had a record present in ECOSS and so had an organism isolated, for any infection type. Of these, 36,482 (30.0%) patients had a urinary *E. coli* isolated, of whom 25,715 (70.5%) had an *E. coli* with resistance to any of the 5 UTI-related antimicrobials. There were 7,485 (0.7% of the total sample) patients identified as developing a blood borne *E. coli* bacteraemia following any UTI-related antimicrobial. Patients who were male, older, more deprived, classed as a long-term user or a treatment failure were more likely to have an *E. coli* bacteraemia reported. A slightly higher proportion of patients prescribed the UTI-specific antimicrobial nitrofurantoin, 2,858 (1.2%), reported a subsequent *E. coli* bacteraemia (Table 2).

**Table 2:** Characteristics of UTI-related antimicrobial users who did / did not have an *E. coli * positive blood culture during study period 2009–2012

		All	Blood culture positive for ***E. coli***	
			No	Yes
	N	1,093,227	1,085,742	7485 (0.7%)
**Gender**				
	Female Male	782,253 (71.6%) 310,974 (28.4%)	777,775 (99.4%) 307,967 (99.0%)	4478 (0.6%) 3007 (1.0%)
**Age category at sample**				
	16-34 35-49 50-64 > 65	268,896 (24.6%) 242,173 (22.2%) 247,287 (22.6%) 334,871 (30.6%)	268,610 (99.9%) 241,693 (99.8%) 246,045 (99.5%) 329,394 (98.4%)	286 (0.1%) 480 (0.2%) 1242 (0.5%) 5477 (1.6%)
**Mean age at sample**				
	Mean(SD)	51.8 (20.6)	51.6 (20.5)	70.9 (15.6)
**SIMD quintile (Q1 = Most Deprived, Q5 = Least Deprived)**				
	Q1 Q2 Q3 Q4 Q5	247,671 (22.8%) 232,451 (21.4%) 215,015 (19.8%) 198,832 (18.3%) 190,210 (17.5%)	245,735 (99.2%) 230,731 (99.3%) 213,604 (99.3%) 197,581 (99.4%) 189,084 (99.4%)	1936 (0.8%) 1720 (0.7%) 1411 (0.7%) 1251 (0.6%) 1126 (0.6%)
**Long-term users of any antimicrobials**				
	No Yes	1,008,191 (92.2%) 85,036 (7.8%)	1,002,338 (99.4%) 83,404 (98.1%)	5853 (0.6%) 1632 (1.9%)
**Treatment failures**				
	No Yes	898,043 (82.1%) 195,184 (17.9%)	892,839 (99.4%) 192,903 (98.8%)	5204 (0.6%) 2281 (1.2%)
**Patients receiving sentinel antimicrobials**				
	Amoxicillin Ciprofloxacin Amoxicillin/clavulanic acid Nitrofurantoin Trimethoprim Excl.Amoxicillin^a^ UTI-Specific^b^	449,099 (41.1%) 260,266 (23.8%) 335,784 (30.7%) 244,612 (22.4%) 669,400 (61.2%) 1,092,151 (99.9%) 752,225 (68.8%)	445,529 (99.2%) 257,583 (99.0%) 333,095 (99.2%) 241,754 (98.8%) 664,343 (99.2%) 1,084,667 (99.3%) 746,421 (99.2%)	3570 (0.8%) 2683 (1.0%) 2689 (0.8%) 2858 (1.2%) 5057 (0.8%) 7484 (0.7%) 5804 (0.8%)

^a^ This includes patients receiving any of ciprofloxacin, amoxicillin/clavulanic acid, nitrofurantoin, trimethoprim

^b^ UTI-specific includes either trimethoprim or nitrofurantoin

Multivariable analysis showed that the stronger predictor for a positive blood culture for *E. coli* bacteraemia was increasing age, along with male gender (OR 1.26, 95% CI 1.20 to 1.33), greater deprivation, increasing age and co-morbidity, and increasing number of UTI specific (trimethoprim or nitrofurantoin) prescriptions in the previous 6 months. There was a 15% increase in the risk of *E. coli* bacteraemia for each additional prescription of a UTI specific antimicrobial dispensed over the previous 6-month period. Emergency hospital admission or admission to a high dependency unit in the previous six months was also associated with a greater likelihood of having a *E. coli* bacteraemia (Table 3).

**Table 3:** Multivariable logistic model for predictors for testing positive for an *E. coli* bacteraemia in relation to UTI-specific drug

	OR	95% CI
Gender (Male) SIMD2 SIMD3 SIMD4 SIMD5 Age (35–49) Age (50–64) Age (> 65) Charl. Index: 1 Charl. Index: 2 Charl. Index: 3 + Number of UTI spec. prescriptions in past 6 months Emergency admissions in prior interval (Yes) Routine admissions in prior interval (Yes) High dependency admissions in prior interval (Yes)	1.26 0.87 0.82 0.77 0.78 1.61 3.41 7.67 2.49 3.64 5.74 1.15 1.24 0.93 1.14	(1.20, 1.33) (0.81, 0.93) (0.76, 0.88) (0.71, 0.83) (0.72, 0.85) (1.35, 1.91) (2.93, 3.97) (6.64, 8.85) (2.27, 2.73) (3.32, 3.99) (5.30, 6.21) (1.13, 1.18) (1.16, 1.32) (0.87, 0.99) (1.01, 1.27)

### Antimicrobial resistance

There were 7,485 patients who were recorded as having an *E. coli* bacteraemia. Characteristics of patients with *E. coli* bacteraemia are detailed in Supplementary Table 1. Resistance rates were high, with 71.2% patients had an *E. coli* that was resistant to at least one of the antimicrobials examined. Resistance was greater for amoxicillin (68.2% of those tested), followed by trimethoprim (44.9%), amoxicillin/clavulanic acid (34.8%), ciprofloxacin (21.1%) and nitrofurantoin (9.8%).

Patients in the most deprived quintile, who were long-term users or treatment failures had higher proportions of resistant *E. coli* bacteraemia. There were 1,313 (80%) long-term users recorded as having a resistant *E. coli* versus 69% of those who were not long-term users (p < 0.001); while 1,765 (76%) of patients who reported a treatment failure had a resistant *E. coli* compared to 69% of those with no treatment failures (p < 0.01). (Supplementary Table 1). Resistance rates were similar across age groups, ranging from 66.2 to 71.6% with no significant difference.

A multivariable model for predictors of resistance in *E. coli* bacteraemia to trimethoprim showed significant predictors were male gender, increasing age, the highest level of deprivation, greater co-morbidity, prior exposure to trimethoprim, a treatment failure with trimethoprim, a sensitive *E. coli* bacteraemia in previous intervals and emergency admission (Table 4). Increasing age and greater co-morbidity were by far the most significant predictor, with a seven-fold likelihood of having a trimethoprim-resistant *E. coli* bacteraemia for those aged 65 years old or older (OR 7.18, 95% CI 5.70 to 9.04), and five-fold for those with a Charlson Index of comorbidity score of 3+ (OR 5.42, 95% CI 4.82 to 6.09).

**Table 4:** Multivariable logistic model for predictors of trimethoprim resistance in *E. coli* bacteraemia isolates

	OR	95% CI
Gender (Male) SIMD2 SIMD3 SIMD4 SIMD5 Age (35–49) Age (50–64) Age (> 65) Charl. Index: 1 Charl. Index: 2 Charl. Index: 3 + Number of trimethoprim prescriptions in last 6 months (per increase of one) Prescribed trimethoprim in previous intervals (Yes) Treatment failure of trimethoprim (Yes) Had sensitive blood test in previous intervals (Yes) Emergency admissions in prior interval (Yes)	1.29 0.83 0.77 0.74 0.86 1.58 3.21 7.18 2.65 3.46 5.42 1.14 1.24 2.03 3.21 1.45	(1.19, 1.40) (0.74, 0.94) (0.68, 0.87) (0.65, 0.84) (0.75, 0.97) (1.20, 2.09) (2.52, 4.10) (5.70, 9.04) (2.31, 3.03) (3.01, 3.97) (4.82, 6.09) (1.04, 1.24) (1.20, 1.28) (1.81, 2.28) (2.44, 4.22) (1.31, 1.60)

A similar model for nitrofurantoin showed the number of prescriptions for nitrofurantoin in the previous 6 months (OR = 1.31, 95% CI 1.10–1.56), and emergency admission (OR = 6.09, 95% CI 2.69–13.79) were also associated with significantly increased risk of resistance to nitrofurantoin in *E. coli* bacteraemia.

### Mortality

Amongst the initial population receiving a UTI-related antibiotic male gender, increasing age, higher levels of deprivation, being a long-term user, having a treatment failure and having an *E. coli* bacteraemia all showed significantly higher proportions of mortality (Table 5).

**Table 5:** Characteristics of all patients prescribed any UTI-related antimicrobial who did/did not die

		All	Died		P-value
			**No**	**Yes**	
	N	1,093,227	1,018,822	74,405 (6.8%)	
**Gender**					
	Female Male	782,253 (71.6%) 310,974 (28.4%)	736,686 (94.2%) 282,136 (90.7%)	45,567 (5.8%) 28,838 (9.3%)	p < 0.001
**Age category at first antimicrobial use**					
	16-34 35-49 50-64 > 65	268,896 (24.6%) 242,173 (22.2%) 247,287 (22.6%) 334,871 (30.6%)	268,286 (99.8%) 239,897 (99.1%) 238,739 (96.5%) 271,900 (81.2%)	610 (0.2%) 2276 (0.9%) 8548 (3.5%) 62,971 (18.8%)	p < 0.001
**Age at first antimicrobial use**					
Mean (SD)51.8 (20.6)49.9 (19.8)77.0 (12.8)p < 0.001					
**SIMD quintile (Q1 = Most Deprived, Q5 = Least Deprived)**					
	Q1 Q2 Q3 Q4 Q5	247,671 (22.8%) 232,451 (21.4%) 215,015 (19.8%) 198,832 (18.3%) 190,210 (17.5%)	230,413 (93.0%) 215,630 (92.8%) 200,047 (93.0%) 185,222 (93.2%) 178,846 (94.0%)	17,258 (7.0%) 16,821 (7.2%) 14,968 (7.0%) 13,610 (6.8%) 11,364 (6.0%)	p < 0.001
**Long-term users of any antimicrobials**					
	No Yes	1,008,191 (92.2%) 85,036 (7.8%)	945,356 (93.8%) 73,466 (86.4%)	62,835 (6.2%) 11,570 (13.6%)	p < 0.001
**At least one treatment failure of any antimicrobials (different antimicrobial within 60 days)**					
	No Yes	898,043 (82.1%) 195,184 (17.9%)	841,648 (93.7%) 177,174 (90.8%)	56,395 (6.3%) 18,010 (9.2%)	p < 0.001
***E. coli*** ** bacteraemia**					
	No Yes	1,085,742 (99.3%) 7485 (0.7%)	1,013,978 (93.4%) 4844 (64.7%)	71,764 (6.6%) 2641 (35.3%)	p < 0.001

In patients who had an *E. coli* bacteraemia tested against trimethoprim, mortality was higher in those that were female, were long-term users of trimethoprim and who were treatment failures (had a second antibiotic in a 60-day period). Mortality in these patients was increased as age increased and was significantly higher in patients with trimethoprim resistance (39.4% v 31.5%, p < 0.001, see Table 6).

**Table 6:** Characteristics of patients with E. coli bacteraemia tested for trimethoprim resistance who did/did not die

		***E. coli*** blood test for trimethoprim resistance	Died		P-value
			No	Yes	
	N	6401	4157 (64.9%)	2244 (35.1%)	
Ever received trimethoprim					
	No Yes	2071 (32.4%) 4330 (67.6%)	1261 (60.9%) 2896 (66.9%)	810 (39.1%) 1434 (33.1%)	p < 0.001
Gender					
	Female Male	3807 (59.5%) 2594 (40.5%)	2603 (68.4%) 1554 (59.9%)	1204 (31.6%) 1040 (40.1%)	p < 0.001
Age category at first trimethoprim use					
	16-34 35-49 50-64 > 65	233 (3.6%) 416 (6.5%) 1052 (16.4%) 4700 (73.4%)	217 (93.1%) 332 (79.8%) 765 (72.7%) 2843 (60.5%)	16 (6.9%) 84 (20.2%) 287 (27.3%) 1857 (39.5%)	p < 0.001
Age at first trimethoprim use					
	Mean (SD)	70.9 (15.6)	71.0 (16.1)	72.8 (14.2)]	p = 0.332
SIMD quintile (Q1 = Most Deprived, Q5 = Least Deprived)					
	Q1 Q2 Q3 Q4 Q5	1625 (25.5%) 1483 (23.3%) 1215 (19.1%) 1052 (16.5%) 989 (15.5%)	1084 (66.7%) 963 (64.9%) 796 (65.5%) 678 (64.4%) 605 (61.2%)	541 (33.3%) 520 (35.1%) 419 (34.5%) 374 (35.6%) 384 (38.8%)	p = 0.071
Long-term users of trimethoprim					
	No Yes	6136 (95.9%) 265 (4.1%)	3968 (64.7%) 189 (71.3%)	2168 (35.3%) 76 (28.7%)	p = 0.030
At least one treatment failure of trimethoprim (different antimicrobial within 60 days); includes prescriptions dispensed before 01/11/2012					
	No Yes	5698 (89.0%) 703 (11.0%)	3678 (64.5%) 479 (68.1%)	2020 (35.5%) 224 (31.9%)	p = 0.065
Resistant					
	No Yes	3523 (55.0%) 2878 (45.0%)	2412 (68.5%) 1745 (60.6%)	1111 (31.5%) 1133 (39.4%)	p < 0.001

## Discussion

### Main findings and comparison with other literature

This is one of the first studies in the UK to use a national linked patient level data set to investigate the population receiving antibiotics for the treatment of UTIs, and has demonstrated an association between primary care prescribing of antimicrobials for UTI and antimicrobial resistance in subsequent *E. coli* bacteraemia.

There were just over 15,000 *E. coli* bacteraemias in Scotland (Health Protection Scotland communication) over the period 2009-12 meaning that almost 50% of all cases came from the initial population within this study. This corresponds to the report by Bhattacharya et al. who made similar estimates for the English population [43].

Our study confirms that most treatment for UTI occurs within the elderly female population, a demographic that is set to significantly increase by 2030 with a 50% projected increase in the number of people aged over 75 years [44]. In our study, of the overall population receiving antibiotics almost 10% were on long-term antimicrobials, presumably as prophylaxis. 7,485 (0.7%) patients initiated on a UTI-related antibiotic had a positive blood culture for *E. coli*, with 71% of this group reported as being resistant to at least one antibiotic. Prior antimicrobial use, along with prior treatment failure, male gender, higher deprivation and multi-morbidity, were associated with a greater likelihood of an *E. coli* bacteraemia with resistance reported in multivariate logistic regression analysis. Increasing age was associated with both a greater likelihood of being prescribed antibiotics, and higher resistance rates. Prior trimethoprim use within the last 12 months was associated with an increased odds for resistance of 20%, with multiple courses showing increased association, which is in line with previous evidence [6]. One-fifth of patients in this national dataset had treatment failures requiring a second different antibiotic to be prescribed within 60 days, with both of the UTI-specific antimicrobials trimethoprim and nitrofurantoin having significant failure rates, of 10% and 16% respectively. Mortality was significantly greater in those with *E. coli* bacteraemia (vs. non-bacteraemic [35.3% vs. 6.6%, p < 0. 001), and those with trimethoprim-resistant *E. coli*. Prior treatment failures were also associated with increased likelihood of mortality, although the relationship was weaker in the case of trimethoprim. Our population with an *E. coli* bacteraemia had just over 35% all-cause mortality during the three-year follow-up and whilst this cannot be directly compared to the 30-day figure in the Bhattacharya paper it is also high. We were unable to examine attributable mortality to *E. coli* or to estimate the excess mortality in this patient group, as we looked into all-cause mortality within the study period modelled at risk over the next 6-months, as opposed to 30-day mortality.

Our findings are consistent with those seen elsewhere. Lishman et al. examined the relationship between primary care prescribing for urinary tract infections and resistance in *E. coli* bacteraemia in adult women in England. Similar to our study, they demonstrated that primary care antibiotic use for UTIs was linked to the development of an UTI-related bacteraemia, with higher rates of resistance associated with prior antimicrobial use [45]. Bou-Anton S et al. examined the incidence, risk factors and antimicrobial susceptibility profile on *E. coli* bacteraemia in England over a two-year period. Increasing age and female gender were both associated with an increased likelihood of *E. coli* bacteraemia, with a large proportion of cases (over 40%) found to be originating from urinary tract infections [34], supporting our findings. Results from Costelloe et al. suggest that primary care prescription of antibiotics was associated with trimethoprim resistance up to 12 months after exposure in patients admitted to hospital with urinary tract infections (unadjusted OR 3.58) [18], and another French study also found that resistance to amoxicillin/clavulanic acid was four times higher with exposure to it in the month before, in patients hospitalised with urinary tract infection [46]. However, the majority of these studies are relatively small, and Lishman included only women, through an ecological, population, approach. We included nationwide Scottish data for all adults from 16 years old and both genders, showing that male gender was a significant factor for resistance. Also, the availability of patient-level data makes it possible to draw patient-level conclusions, avoiding ecological bias. O Blandy et al. also demonstrated that presence of antimicrobial resistance, increasing age and comorbidities as main contributing factors for increased mortality [35], similar to our results, with the majority of cases being of community-onset, highlighting the need for improving antimicrobial use and reduce the burden of *E. coli* infections in the community. Similarly, increasing co-morbidity and an adverse antimicrobial resistance profile have been described as risk factors for poorer outcomes in studies looking more widely at Gram-negative bacteraemias [47].

### Implications for policy and practice

There were over 1 million adult patients receiving at least one prescription for the 5 UTI-related antimicrobials within the study period, which means that 24.9% of the Scottish population (based on mid-2011 estimates of numbers aged 16–95+) [48] were exposed to these antimicrobials in this time frame. Primary care antimicrobial exposure was associated with increased occurrence of resistance in *E. coli* bloodstream infections (particularly for trimethoprim or nitrofurantoin), which in turn was associated with increased mortality. Our findings reinforce national policy efforts trying to reduce total antimicrobial use in the community in Scotland [49], and particularly antibiotic use in urinary tract infections, similar to the English Quality Premium initiative to reduce Gram-negative bloodstream infections [50]. Improving surveillance of resistance has also been described as a key action point for both UK Government [51], and internationally [52]. This study has helped demonstrate the value of enhanced surveillance systems, such as ECOSS, and routine data linkage for monitoring resistance trends nationally, understanding precipitating factors (including primary care antibiotic use), and examining adverse outcomes, such as mortality.

Both UTI-related antimicrobials had significant failure rates. This will have considerable resource and patient outcome implications, including longer hospital stays, increased mortality, and increased cost related to suboptimal antimicrobial use [53–55]. Furthermore, this has implications for the recommendations for empiric therapy of UTIs. The majority of prescribing is done without knowledge of the infecting organism or its sensitivities as, although laboratory reporting can influence GPs’ prescribing of antibiotics for UTIs and other infections [56], current guidelines recommend against routine sampling for uncomplicated urinary tract infections in the community [30].

## Conclusion

Increasing age, increasing co-morbidity, lower socioeconomic status, and prior community antibiotic exposure were significantly associated with a resistant *E. coli* bacteraemia, which leads to increased mortality, with older age being the strongest predictor. This highlights the need for prudent primary care antibiotic use, particularly in the frail, multi-morbid population.

## Strengths & Limitations

The major strength of this study is that we report data on the burden of antimicrobial resistance in *E. coli* bacteraemia in Scotland at national level, and the association with prior antimicrobial use for UTI in the community among other factors, and mortality at the patient-level, which is unique. Completeness is a common concern with using routinely collected data. However, the data presented in this study derived from the ECOSS data set, which entails a surveillance and reporting system across Scotland. Reporting of *E. coli* bacteraemia to ECOSS remains mandatory, so missing data is unlikely.

Patients were identified as having an antimicrobial prescription of any of the five specified antimicrobials at any point during the study period. Patients having other or no antimicrobials but still an *E. coli* bacteraemia could have been missed. The five antimicrobials examined however represent up to 80% antimicrobial use for UTI in the community, and are all relevant for the treatment of Gram-negative bacteraemia [23]. However, we did not have the indication for the prescription as this is not available within the PIS database, which may introduce selection bias, and so assumed that UTI was the reason for the prescription. This is more valid for trimethoprim and nitrofurantoin but less so for the other antimicrobials especially amoxicillin and so we presented data grouped in different ways.

We defined treatment failure as needing a different, second, antimicrobial within 60 days (which is longer than some other studies [23]), as we assumed this was used to treat the same infection. We acknowledge this is a strong assumption, however we decided to use a 60-day window to identify treatment failure as not all PIS data has the correct dispensed date attached as it may default to the date the pharmacist was paid which is commonly the end of the month. We therefore chose a 60-day window to allow for this limitation with the data. There is also the issue that treatment failure is actually empirical treatment of an existing undiagnosed resistant *E. coli* which is only detected at a later stage, but we were unable to explore the data to examine this further and it also reflects clinical practice.

## Electronic supplementary material

Below is the link to the electronic supplementary material.

Supplementary Material 1

## Data Availability

The data that support the findings of this study are available to named researchers from the Electronic Data Research and Innovation Service (eDRIS) at Public Health Scotland, but restrictions apply to the availability of these data, which were used under license for the current study, and so are not publicly available.

## Declarations

The study was an observational study performing secondary data analysis of routinely collected health data. No experiments or interventions on humans were performed as part of the study. The research team did not collect any human samples. The study analyses anonymised information from blood cultures collected as part of routine healthcare practice.

Analysis of anonymised information was done as per UK Good Clinical Practice guidelines, in a secure, password-protected Safe Haven part of Information Services Division (eDRIS) at Public Health Scotland, under NHS Scotland Research Ethics approval as detailed above.

## List of abbreviations

AMR: Antimicrobial resistance
CHI: community health index number
CI: Confidence interval
CLSI: Clinical and Laboratory Standard Institute
DDDs: Defined Daily Doses
*E. coli*: *Escherichia coli*
ECOSS: Electronic Communication of Surveillance in Scotland (microbiology dataset)
eDRIS: electronic Data Research and Innovation Service
ICD-10: International Classification of Diseases
NHS: National Health System
NRS: National Registrar Scotland (mortality dataset)
NSS: NHS National Services Scotland
OR: Odds Ratio
PAC: Privacy Advisory Committee
PIS: Prescribing Information System (prescribing dataset)
SMR01: NSS General/Acute Inpatient and Day Case (hospital admission dataset)
UTI: Urinary tract infection(s)

## References

[1] Morgan MG, McKenzie H (1993). Controversies in the laboratory diagnosis of community-acquired urinary tract infection. Eur J Clin Microbiol Infect diseases: official publication Eur Soc Clin Microbiol.

[2] Tackling drug-resistant. infections globally: final report and recommendations. The review on antimicrobial resistance. O’Neill J. May 16th 2016. https://amr-review.org/sites/default/files/160525_Final%20paper_with%20cover.pdf (Last accessed 9th April 2019).

[3] Cheng ACTJ, Collington P, Looke D, Barton M, Gottlieb T (2012). Control of fluoroquinolone resistance through successful regulation, Australia. Emerg Infect Dis.

[4] Magee JT, Pritchard EL, Fitzgerald KA, Dunstan FD, Howard AJ. Antibiotic prescribing and antibiotic resistance in community practice: retrospective study, 1996-8. BMJ (Clinical research ed). 1999;319(7219):1239–40. 10.1136/bmj.319.7219.1239 PMC28274 10550088

[5] Priest P, Yudkin P, McNulty C, Mant D (2001). Antibacterial prescribing and antibacterial resistance in English general practice: cross sectional study. BMJ.

[6] Costelloe C, Metcalfe C, Lovering A, Mant D, Hay AD (2010). Effect of antibiotic prescribing in primary care on antimicrobial resistance in individual patients: systematic review and meta-analysis. BMJ (Clinical research ed).

[7] Bell BG, Schellevis F, Stobberingh E, Goossens H, Pringle M (2014). A systematic review and meta-analysis of the effects of antibiotic consumption on antibiotic resistance. BMC Infect Dis.

[8] Donnan PT, Wei L, Steinke DT, Phillips G, Clarke R, Noone A (2004). Presence of bacteriuria caused by trimethoprim resistant bacteria in patients prescribed antibiotics: multilevel model with practice and individual patient data. BMJ (Clinical research ed).

[9] Hillier S, Roberts Z, Dunstan F, Butler C, Howard A, Palmer S (2007). Prior antibiotics and risk of antibiotic-resistant community-acquired urinary tract infection: a case-control study. J Antimicrob Chemother.

[10] Seppala H, Klaukka T, Vuopio-Varkila J, Muotiala A, Helenius H, Lager K (1997). The effect of changes in the consumption of macrolide antibiotics on erythromycin resistance in group A streptococci in Finland. Finnish Study Group for Antimicrobial Resistance. N Engl J Med.

[11] Austin DJ, Kristinsson KG, Anderson RM (1999). The relationship between the volume of antimicrobial consumption in human communities and the frequency of resistance. Proc Natl Acad Sci USA.

[12] Butler CC, Dunstan F, Heginbothom M, Mason B, Roberts Z, Hillier S (2007). Containing antibiotic resistance: decreased antibiotic-resistant coliform urinary tract infections with reduction in antibiotic prescribing by general practices. Br J Gen practice: J Royal Coll Gen Practitioners.

[13] Sundqvist M, Geli P, Andersson DI, Sjolund-Karlsson M, Runehagen A, Cars H (2010). Little evidence for reversibility of trimethoprim resistance after a drastic reduction in trimethoprim use. J Antimicrob Chemother.

[14] Enne VI, Livermore DM, Stephens P, Hall LM (2001). Persistence of sulphonamide resistance in Escherichia coli in the UK despite national prescribing restriction. Lancet.

[15] Holmes AH, Moore LSP, Sundsfjord A, Steinbakk M, Regmi S, Karkey A (2016). Understanding the mechanisms and drivers of antimicrobial resistance. The Lancet.

[16] Brolund A, Sundqvist M, Kahlmeter G, Grape M (2010). Molecular characterisation of trimethoprim resistance in Escherichia coli and Klebsiella pneumoniae during a two year intervention on trimethoprim use. PLoS ONE.

[17] Duffy MA, Hernandez-Santiago V, Orange G, Davey PG, Guthrie B (2013). Trimethoprim prescription and subsequent resistance in childhood urinary infection: multilevel modelling analysis. Br J Gen practice: J Royal Coll Gen Practitioners.

[18] Costelloe C, Williams MO, Montgomery AA, Dayan C, Hay DA. Antibiotic Prescribing in Primary Care and Antimicrobial Resistance in Patients Admitted to Hospital with Urinary Tract Infection: A Controlled Observational Pilot Study. Antibiotics. 2014;3(1). 10.3390/antibiotics3010029 PMC4790346 27025731

[19] Vellinga A, Tansey S, Hanahoe B, Bennett K, Murphy AW, Cormican M (2012). Trimethoprim and ciprofloxacin resistance and prescribing in urinary tract infection associated with Escherichia coli: a multilevel model. J Antimicrob Chemother.

[20] Malcolm W, Fletcher E, Kavanagh K, Deshpande A, Wiuff C, Marwick C (2018). Risk factors for resistance and MDR in community urine isolates: population-level analysis using the NHS Scotland Infection Intelligence Platform. J Antimicrob Chemother.

[21] Fleming-Dutra KE, Hersh AL, Shapiro DJ (2016). Prevalence of inappropriate antibiotic prescriptions among us ambulatory care visits, 2010–2011. JAMA.

[22] Shapiro DJ, Hicks LA, Pavia AT, Hersh AL (2014). Antibiotic prescribing for adults in ambulatory care in the USA, 2007–09. J Antimicrob Chemother.

[23] Ahmed H, Farewell D, Jones HM, Francis NA, Paranjothy S, Butler CC (2018). Incidence and antibiotic prescribing for clinically diagnosed urinary tract infection in older adults in UK primary care, 2004–2014. PLoS ONE.

[24] Primary Care Adult Empirical Treatment of Infection Guidance. NHS Tayside. Last updated March 2021 (accessed on 13th. May 2022) [Available from: https://www.nhstaysideadtc.scot.nhs.uk/antibiotic%20site/pdf%20docs/Antibiotic%20primary%20care%20man.pdf.

[25] NHS Lothian Joint Formulary - Therapeutic Areas - Infection - Urinary tract. (last accessed 13th. May 2022) [Available from: https://formulary.nhs.scot/east/infections/urinary-tract/.

[26] NHS Fife Area Drugs and Therapeutics Committee. Adult Primary Care Antibiotic Guidelines (last accessed 13th May 2022). [Available from: https://www.fifeadtc.scot.nhs.uk/formulary/5-infections/adult-primary-care-antibiotic-guidelines.aspx.

[27] Scottish Intercollegiate Guidelines Network (SIGN). Management of suspected bacterial lower urinary tract infection in adult women. Edinburgh: SIGN; 2020. (SIGN publication no. 160). [September 2020]. Available from URL: http://www.sign.ac.uk (last accessed 13th May 2022) [Available from: https://www.sign.ac.uk/our-guidelines/management-of-suspected-bacterial-lower-urinary-tract-infection-in-adult-women/.

[28] Scottish Antimicrobial Prescribing Group, Healthcare Improvement Scotland. Infection-specific guidance: Urinary tract infections (last accessed 13th. May 2022) [Available from: https://www.sapg.scot/guidance-qi-tools/infection-specific-guidance/urinary-tract-infections/.

[29] Health Protection Scotland. Scottish National Point Prevalence Survey of Healthcare Associated Infection and Antimicrobial Prescribing 2016. Health Protection Scotland. 2017.

[30] Scottish Intercollegiate Guidelines Network (SIGN). Management of suspected bacterial urinary tract infection in adults. Edinburgh: SIGN; 2012 (SIGN publication no 88). 2012.

[31] National Institute for Health and Care Excellence. Urinary tract infection (lower): antimicrobial prescribing NG109 [Internet]. London: NICE; 2018 [last accessed 2022 May 13]. Available from: https://www.nice.org.uk/guidance/ng80.

[32] Melzer M, Petersen I (2007). Mortality following bacteraemic infection caused by extended spectrum beta-lactamase (ESBL) producing E. coli compared to non-ESBL producing E. coli. J Infect.

[33] Friedman ND, Temkin E, Carmeli Y (2016). The negative impact of antibiotic resistance. Clin Microbiol Infect.

[34] Bonten M, Johnson JR, van den Biggelaar AHJ, Georgalis L, Geurtsen J, de Palacios PI (2021). Epidemiology of Escherichia coli Bacteremia: A Systematic Literature Review. Clin Infect Dis.

[35] Blandy O, Honeyford K, Gharbi M, Thomas A, Ramzan F, Ellington MJ (2019). Factors that impact on the burden of Escherichia coli bacteraemia: multivariable regression analysis of 2011–2015 data from West London. J Hosp Infect.

[36] Alvarez-Madrazo S, McTaggart S, Nangle C, Nicholson E, Bennie M (2016). Data Resource Profile: The Scottish National Prescribing Information System (PIS). Int J Epidemiol.

[37] Scottish Government. Scottish Index of Multiple Deprivation: Scottish Government; 2012 [Available from: http://www.scotland.gov.uk/Topics/Statistics/SIMD.

[38] https://www.isdscotland.org/Products-and-Services/eDRIS/.

[39] Charlson ME, Pompei P, Ales KL, MacKenzie CR (1987). A new method of classifying prognostic comorbidity in longitudinal studies: development and validation. J chronic Dis.

[40] ISO 20776-2E Clinical. laboratory testing and in vitro diagnostic test systems — Susceptibility testing of infectious agents and evaluation of performance of antimicrobial susceptibility test devices — Part 2.

[41] Efron B, Logistic, Regression (1988). Survival Analysis, and the Kaplan-Meier Curve. J Am Stat Assoc.

[42] R Core Team. R: A language and environment for statistical computing. R Foundation for Statistical Computing, Vienna, Austria URL https://wwwR-projectorg. 2017.

[43] Bhattacharya A, Nsonwu O, Johnson AP, Hope R (2018). Estimating the incidence and 30-day all-cause mortality rate of Escherichia coli bacteraemia in England by 2020/21. J Hosp Infect.

[44] National Records of Scotland. (2017) Projected Population of Scotland (2016-based), National Records of Scotland, Edinburgh. https://www.nrscotland.gov.uk/files//statistics/population-projections/2016-based-scot/pop-proj-2016-scot-nat-pop-pro-pub.pdf.

[45] Lishman H, Costelloe C, Hopkins S, Johnson AP, Hope R, Guy R, et al. Exploring the relationship between primary care antibiotic prescribing for urinary tract infections, Escherichia coli bacteraemia incidence and antibiotic resistance: an ecological study. International Journal of Antimicrobial Agents. 10.1016/j.ijantimicag.2018.08.013 30145249

[46] Heym B, Ternat G, Nicolas-Chanoine M-H, Leflon-Guibout V (2002). Exposure to co-amoxiclav as a risk factor for co-amoxiclav-resistant Escherichia coli urinary tract infection. J Antimicrob Chemother.

[47] Baltas I, Stockdale T, Tausan M, Kashif A, Anwar J, Anvar J (2021). Long-term outcome and risk factors for late mortality in Gram-negative bacteraemia: a retrospective cohort study. J Global Antimicrob Resist.

[48] National Records of Scotland. Detailed Data Zone Tables - Mid 2011. https://www.nrscotland.gov.uk/statistics-and-data/statistics/statistics-by-theme/population/population-estimates/2011-based-special-area-population-estimates/small-area-population-estimates/mid-2011-to-mid-2014/detailed-data-zone-tables-mid-2011.

[49] National Therapeutics Indicatiors. 2018. NHS Scotland. http://www.therapeutics.scot.nhs.uk/wp-content/uploads/2018/08/National-Therapeutic-Indicators-Report-2018-19-Version-1.0.pdf.

[50] NHS England Quality Premium. guidance 2017-19 on reducing gram negative blood stream infections (BSI) across the whole health economy – Part B. https://www.england.nhs.uk/publication/part-a-reducing-gram-negative-blood-stream-infections-bsi-across-the-whole-health-economy/.

[51] UK 5-year. action plan for antimicrobial resistance 2019–2024. https://www.gov.uk/government/publications/uk-5-year-action-plan-for-antimicrobial-resistance-2019-to-2024.

[52] Global action plan on antimicrobial resistance. World Health Organisation, 2015.

[53] Abernethy JK, Johnson AP, Guy R, Hinton N, Sheridan EA, Hope RJ. Thirty day all-cause mortality in patients with Escherichia coli bacteraemia in England. Clinical microbiology and infection: the official publication of the European Society of Clinical Microbiology and Infectious Diseases. 2015;21(3):251.e1-8. 10.1016/j.cmi.2015.01.001 25698659

[54] Ng E, Earnest A, Lye DC, Ling ML, Ding Y, Hsu LY. The excess financial burden of multidrug resistance in severe gram-negative infections in Singaporean hospitals. Ann Acad Med Singap. 2012;41. 22760715

[55] Peralta G, Sanchez MB, Garrido JC, De Benito I, Cano ME, Martinez-Martinez L (2007). Impact of antibiotic resistance and of adequate empirical antibiotic treatment in the prognosis of patients with Escherichia coli bacteraemia. J Antimicrob Chemother.

[56] McNulty CA, Lasseter GM, Charlett A, Lovering A, Howell-Jones R, Macgowan A (2011). Does laboratory antibiotic susceptibility reporting influence primary care prescribing in urinary tract infection and other infections?. J Antimicrob Chemother.

