# Supplementary material for: E. coli bacteraemia and antimicrobial resistance following antimicrobial prescribing for urinary tract infection in the community
Source: BMC Infect Dis. 2022 Oct 28;22:805. doi: 10.1186/s12879-022-07768-7 (PMC9621144; doi:10.1186/s12879-022-07768-7)
Supplement: Supplementary file 1 — Supplementary Material 1 [file 12879_2022_7768_MOESM1_ESM.docx]

| **Supplementary Table 1: Characteristics of patients with a positive blood culture for E.coli** **comparing resistant *versus* sensitive isolates** | | | | | |
| --- | --- | --- | --- | --- | --- |
|  |  |  | ***E.coli* bacteraemia isolate ever shown resistance to one of the five sentinel antimicrobials on culture** | | **P-value** |
|  |  |  | **No** | **Yes** |  |
|  | N | 7485 | 2158 | 5327 (71.2%) |  |
| **Gender** | | | | | |
|  | Female Male | 4478 (59.8%)  3007 (40.2%) | 1276 (28.5%)  882 (29.3%) | 3202 (71.5%)  2125 (70.7%) | p=0.449 |
| **Age category at first antimicrobial use** | | | | | |
|  | 16-34 35-49 50-64 >65 | 286 (3.8%)  480 (6.4%)  1242 (16.6%)  5477 (73.2%) | 84 (29.4%)  162 (33.8%)  359 (28.9%)  1553 (28.4%) | 202 (70.6%)  318 (66.2%)  883 (71.1%)  3924 (71.6%) | p=0.097 |
| **SIMD quintile (Q1= Most Deprived, Q5=Least Deprived)** | | | | | |
|  | Q1 Q2 Q3 Q4 Q5 | 1936 (26.0%)  1720 (23.1%)  1411 (19.0%)  1251 (16.8%)  1126 (15.1%) | 474 (24.5%)  518 (30.1%)  448 (31.8%)  391 (31.3%)  314 (27.9%) | 1462 (75.5%)  1202 (69.9%)  963 (68.2%)  860 (68.7%)  812 (72.1%) | p<0.001 |
| **Long-term users** | | | | | |
|  | No Yes | 5853 (78.2%)  1632 (21.8%) | 1839 (31.4%)  319 (19.5%) | 4014 (68.6%)  1313 (80.5%) | p<0.001 |
| **At least one treatment failure (different antimicrobial within 60 days** | | | | | |
|  | No Yes | 5204 (69.5%)  2281 (30.5%) | 1604 (31.0%)  554 (23.9%) | 3562 (69.0%)  1765 (76.1%) | p<0.001 |
| **Antimicrobial(s) received** | | | | | |
|  | Amoxicillin Ciprofloxacin Co-amoxiclav Nitrofurantoin Trimethoprim Excl.Amoxicillin UTI-Specific | 3570 (47.7%)  2683 (35.8%)  2689 (35.9%)  2858 (38.2%)  5057 (67.6%)  7484 (100.0%) 5804 (77.5%) | 937 (26.2%)  713 (26.6%)  701 (26.1%)  686 (24.0%)  1390 (27.5%)  2158 (28.8%)  1591 (27.4%) | 2633 (73.8%)  1970 (73.4%)  1988 (73.9%)  2172 (76.0%)  3667 (72.5%)  5326 (71.2%)  4213 (72.6%) |  |
|  | **Antimicrobial(s)tested** | | | | |
|  | Amoxicillin Ciprofloxacin Co-amoxiclav Nitrofurantoin Trimethoprim | 6393 (85.4%)  6841 (91.4%)  6976 (93.2%)  1290 (17.2%)  6401 (85.5%) | 1710 (26.7%)  1916 (28.0%)  1916 (27.5%)  365 (28.3%)  1771 (27.7%) | 4683 (73.3%)  4925 (72.0%)  5060 (72.5%)  925 (71.7%)  4630 (72.3%) |  |
